# Mucocele of the Lower Lip and Its Surgical Management

**DOI:** 10.7759/cureus.70874

**Published:** 2024-10-05

**Authors:** Neha Kannan, Karthikeyan Ramalingam, Pratibha Ramani, Murugesan Krishnan

**Affiliations:** 1 Oral Pathology and Microbiology, Saveetha Dental College and Hospitals, Saveetha Institute of Medical and Technical Sciences, Saveetha University, Chennai, IND; 2 Oral and Maxillofacial Surgery, Saveetha Dental College and Hospitals, Saveetha Institute of Medical and Technical Sciences, Saveetha University, Chennai, IND

**Keywords:** adolescent, labial mucosa, lip swelling, lower lip, lower lip swelling, minor salivary glands, mucocele, mucous extravasation cyst, mucous extravasation phenomenon, surgery

## Abstract

We report a case of mucocele of the lower lip in a 17-year-old female patient. She complained of a painless swelling on her lower lip for the last one month. The patient also gave a history of lip-biting. Upon clinical inspection, a soft, round, dome-shaped bluish lesion with a pearly appearance was identified on the right lower labial mucosa. On palpation, the lesion was mobile and non-adherent to the underlying tissues. An excisional biopsy was performed under local anesthesia, and the excised sample was sent for histopathological evaluation. Histopathology revealed mucus pooling, surrounded by compressed connective tissue stroma and areas of chronic inflammation indicative of a mucocele. The post-excisional review was done after 10 days, and the patient’s healing was satisfactory. The patient had no signs of recurrence after two years of follow-up.

## Introduction

Mucoceles is the most commonly encountered disease associated with the minor salivary glands in the oral cavity. They are one of the most common biopsied oral lesions in pediatric and adolescent populations [1]. These benign lesions, characterized by the accumulation of mucus, are classified as extravasation and retention types. The extravasation type refers to the fact that they result from the leakage of saliva into surrounding tissues rather than forming a true cystic lining [1,2]. 

Mucoceles predominantly occur on the lower lip, particularly in children and young adults, often following trauma or irritation to the minor salivary glands [2]. Despite their benign nature, their tendency to fluctuate in size and potentially recur makes them a significant clinical consideration in younger populations. Prompt diagnosis and appropriate management are crucial to avoid discomfort and functional difficulties. Depending on the size and persistence of the lesion, the standard course of treatment is either simple monitoring and follow-up or surgical excision [3].

In this case report, we present mucocele of the lower lip in an adolescent patient. We further discuss the clinical findings, histopathological findings, and the surgical management of the lesion.

## Case presentation

A 17-year-old female patient reported to the outpatient department of Saveetha Dental College and Hospitals with a chief complaint of swelling on her right lower lip for the past one month. The patient gave no history of pain or discomfort, although she had noticed a gradual increase in the size of the swelling over three weeks. She further stated that she bit her lower lip frequently, especially when she was studying.

Past medical history, drug history, surgical history, and dental history were noncontributory. A thorough systemic assessment including the neurological, musculoskeletal, pulmonary, digestive, and cardiovascular systems. Systemic symptoms like fever, weight loss, sweats at night, exhaustion, or changes in appetite were not reported. A comprehensive head and neck examination revealed no lymphadenopathy, growth, or tenderness. Extraoral examination showed that facial symmetry, temporomandibular joint, and muscle function were unaltered.

**Figure 1:**
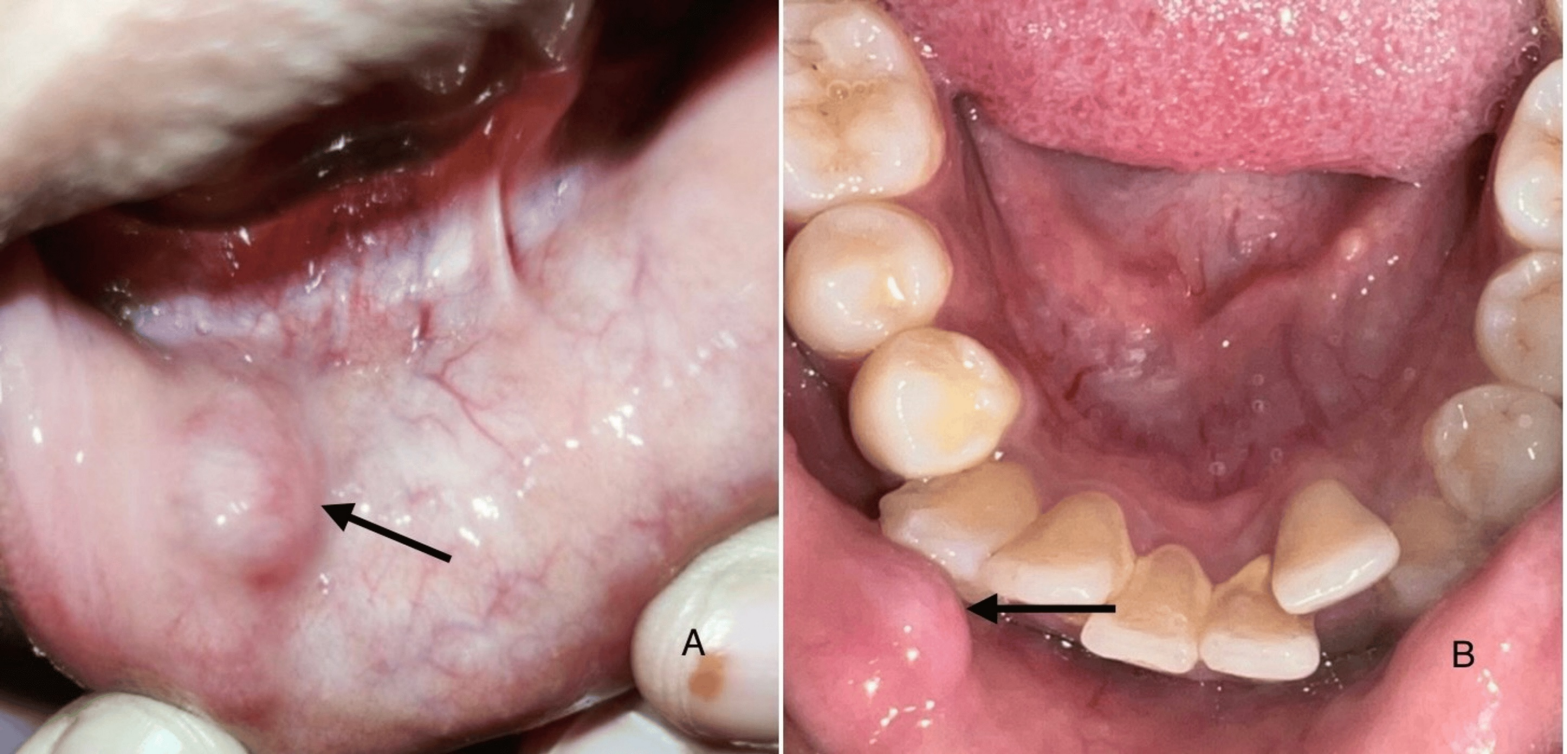
Clinical pictures (A) Clinical picture showing a well-defined, soft dome-shaped swelling on the right labial mucosa; (B) Occlusal view of the lesion related to the incisal edge of the mandibular anterior teeth

On clinical examination, a well-defined, soft, dome-shaped swelling measuring about 1.4 x 1.1 cm was identified on the right lower labial mucosa in relation to the occlusal plane of 42 and 43. Upon palpation, the lesion was mobile and non-adherent to the underlying tissues. The surface of the swelling had a blue-colored tint with a pearly to semi-clear appearance. The overlying mucosa was intact without surface ulcerations or secondary infections (Figure 1).

**Figure 2:**
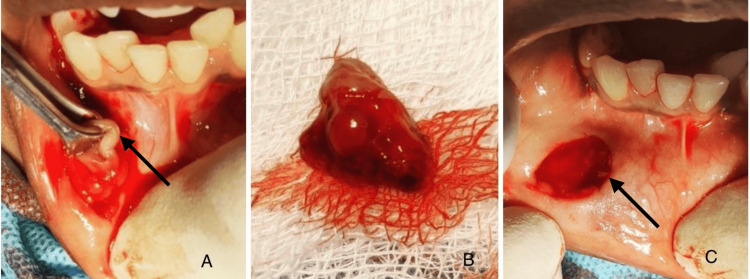
Clinical pictures (A) Intraoperative picture where the swelling is held with Ellis forceps; (B) Excised specimen along with the associated minor salivary gland; (C) Surgically excised site

Given the distinctive clinical presentation and location of the swelling, the provisional diagnosis was given as mucocele. An excisional biopsy was planned for a definitive diagnosis. The management began with the administration of local anesthesia (2 ml of Lignocaine and Adrenaline), carefully infiltrated around the lesion to ensure complete numbness of the area. Once anesthetized, an incision was made over the swelling. Blunt dissection followed, allowing full exposure of the lesion and the associated minor salivary glands. Using Ellis forceps, the lesion was gently held to maintain a grasp, making it easier to identify its point of origin. The entire lesion was excised along with the associated minor salivary gland to prevent recurrence (Figure 2). The wound was then closed using 3-0 silk sutures, employing a simple interrupted suturing technique to ensure proper wound approximation and healing.

**Figure 3:**
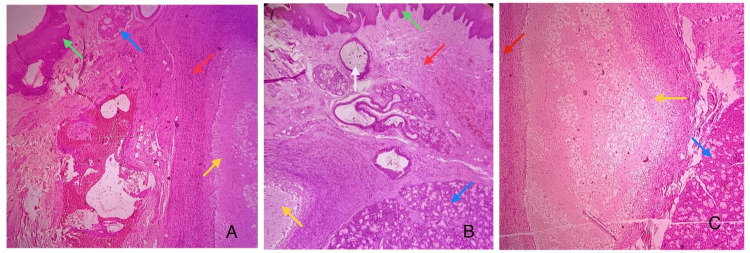
Photomicrographs showing mucin pooling, surface epithelium and minor salivary glands (H&E, 10x) (A) shows surface epithelium (green arrow), minor salivary glands (blue arrow), compressed connective tissue (red arrow), and mucin pooling (yellow arrow); (B) shows surface epithelium (green arrow), dilated ducts (white arrow), minor salivary glands (blue arrow), and mucin pooling (yellow arrow); (C) shows compressed connective tissue (red arrow), mucin pooling (yellow arrow), and minor salivary glands (blue arrow). The green arrow depicts the parakeratinized stratified squamous epithelium; the red arrow depicts the compressed connective tissue with moderate vascularity and inflammatory infiltrate; the yellow arrow depicts the areas of mucin pooling with mucinophages; the blue arrow points towards the mixed salivary gland structures; and the white arrow shows dilated ducts with mucin pooling.

Histopathological examination showed fibrous connective stroma with mucin pooling and abundant mucinophages surrounded by compressed connective tissue and areas of granulation tissue (Figure 3). There was evidence of chronic inflammatory cell infiltrate, moderate vascularity, and areas of hemorrhage. Numerous mucous salivary gland acini with dilated ducts were also seen. Skeletal muscle and adipose tissue were present in the deeper planes. The surface epithelium was parakeratinized stratified squamous epithelium in nature (Figure 3).

The histopathological findings were consistent with a mucocele - mucous extravasation type. The patient was recalled after 10 days for post-excisional review, and the healing was satisfactory. The lesion was monitored for two years after excision, and the patient showed no signs of recurrence.

## Discussion

Two distinct types of mucoceles can occur in the oral cavity: extravasation and retention types [4]. Extravasation mucoceles are frequently observed in children, while retention mucoceles are rarely found in this age group. The extravasation type develops when a salivary gland duct ruptures, allowing mucus to leak into the surrounding soft tissues, thereby forming a pseudocyst [5]. In contrast, retention mucocele is less common and is caused by the obstruction of the salivary gland duct. This leads to mucus accumulation within the duct itself and reduced or absent glandular secretion [6]. 

Both types share a similar pathogenesis, where mucus buildup leads to swelling, typically in the lower lip, although other oral sites may also be affected. These mucoceles go through three stages of development. In the initial phase, mucus spreads from the damaged duct into the connective tissue. The next stage, known as the resorption phase, involves a foreign body reaction with the formation of a granuloma. Finally, a pseudocapsule (without epithelial lining) forms around the affected area [7].

The most frequent cause of a mucocele is trauma, such as accidental biting of the lip or cheek, which can damage or rupture the salivary gland duct, leading to the accumulation of mucin in the surrounding tissues and the formation of a cyst-like structure [8]. Chronic irritation, such as that caused by sharp teeth or dental appliances, can also contribute to the development of a mucocele [9]. Orthodontic issues like crowding, deep bites, or malaligned teeth cause repetitive trauma or compression to the salivary glands [10]. Less common causes include salivary gland duct obstruction due to factors like sialolithiasis (salivary stones), which causes backflow and extravasation of mucus [11].

In this present case, lip swelling prompted a comprehensive diagnostic approach. The evaluation began with a detailed patient history, followed by a thorough clinical examination to assess the lesion's characteristics [12]. Diagnostic procedures included visual inspection and palpation followed by a biopsy to arrive at an accurate diagnosis. This systematic approach ensured a prompt diagnosis and management, adhering to standard protocols for lip swellings in adolescents [13].

In this case, the patient's habitual lip biting during stressful periods, especially while studying, combined with the presence of lower anterior dental crowding, could have caused chronic trauma and played a significant role in the development of a mucocele. It is reported in the literature that dental crowding could have exacerbated the situation by contributing to abnormal occlusal forces or abnormal lip contact, increasing the likelihood of repetitive trauma [14].

The management of mucoceles typically involves surgical excision, especially for persistent or recurrent cases. In some cases, less invasive methods like cryotherapy, laser excision, or marsupialization may be considered, particularly for smaller lesions [15]. Conservative observation is an option for very small, asymptomatic mucoceles that may resolve on their own [16,17]. Our patient had undergone complete excision and is remaining disease-free on follow-up.

Differential diagnoses that should be considered while dealing with oral mucocele are epidermoid cyst, dermoid cyst, lymphoepithelial cyst, lingual cyst, cystic schwannoma, neurofibroma, and minor salivary gland tumors [18]. We excluded these entities by classic histopathology of the mucous extravasation type noted in our case.

## Conclusions

In conclusion, mucoceles are benign lesions commonly arising from minor salivary gland ductal obstruction or trauma, particularly in the lower lip. Their pathogenesis involves a complex interplay of inflammatory responses, which can influence their development and recurrence. Effective management typically requires surgical excision to prevent persistence or reoccurrence.
